# E-Tools for Personalizing Learning During the Pandemic: Case Study of an Innovative Solution for Remote Teaching

**DOI:** 10.3389/fpsyg.2022.751316

**Published:** 2022-05-06

**Authors:** Loredana Adriana Patrascoiu, Ruxandra Folostina, Dan Patzelt, Maria Paula Blaj, Bianca Poptean

**Affiliations:** ^1^Faculty of Psychology and Education Sciences, University of Bucharest, Bucharest, Romania; ^2^Urban Development Association, Bucharest, Romania

**Keywords:** remote teaching, innovative teaching solution, inclusion, e-learning tools, learning design

## Abstract

“Every child counts” has lost its value even from the political discourse of some societies during the pandemic, proving that the level of culture of inclusion is the true standard of Sustainable Development Goal (SDG) commitment. Online education and therapy required rethinking the way we educate children with special needs and, implicitly, prepare them for life. We consider that the personalized approach of the therapeutic intervention was the main difficulty. In this article, we propose a solution to this problem, an approach based on a platform initially developed by tactileimages.org for vision-impaired pupils which became a tool in the universal design of learning materials. This e-learning tool includes an Editor, a browser-based software developed to allow the creation or adaptation of drawings into vector images; the QR code through which areas of educational and therapeutic interest are allocated to pictures for task personalization; and the voice-over function of the companion application. The customized material is identified by image recognition algorithms, and the user's gesture is recognized by artificial intelligence algorithms, which receives (by voice-over) details about therapeutic tasks in remote teaching. The article illustrates the personalization of the therapeutic and educational path. The process starts with defining the child's functioning profile and matching function with the curriculum elements as they are found within the Erasmus + project “Cognitive Resources for Toddlers Teens and Experts” —stored in the virtual library. Information and comunication technology is currently an important vector in attaining the SDG vision. The proposed solution will be improved in order to further personalize the educational and therapeutic intervention in the post-pandemic period too.

## 2030 Agenda for Sustainable Development Goal and The Pre-Pandemic Educational Context

All the 17 goals of the 2030 Agenda for Sustainable Development Goal (SDG) need to be achieved by education involvement, even if Goal 4 is about how we see education in the future. Goal 4 is about ensuring inclusiveness and a good level of education by promoting lifelong learning opportunities for all. The transformative intentions of the 2030 Agenda gain sustainability in the light of a matrix of indicators for immediate, medium, and long-term implementation of the SDGs as Lee et al. (2016) recommend for violence and social issues.

In the context of the Incheon Declaration and the 2030 Agenda, inclusion implies that each person has an equal and personalized chance for educational progress. This has proven to be a social challenge in almost all countries. Despite the progress made over the past two decades to expand access to basic education, further directions are needed to minimize barriers to learning, thus ensuring that all students in schools and other learning environments benefit from a genuine, inclusive environment (International Bureau of Education-UNESCO (IBE-UNESCO), 2016, 2017). We cannot build inclusive and equitable societies if we do not start this process within inclusive education systems.

During the pre-pandemic period, the main difficulty of inclusive education was to personalize the educational and therapeutic approach to create learning environments that generate meaningful learning experiences for each beneficiary (Cantor et al., 2019). This challenge was mirrored in terms of curriculum development and assessment systems that must consider all the learners.

Elaborated before knowing the challenges of the pandemic, “Cognitive Resources for Toddlers Teens and Experts—CORTTEX” is an Erasmus + project meant to training Romanian specialists in cognitive education and neuro-didactics. Developed and implemented by the Department of Psychology of the Faculty of Psychology and Educational Sciences, University of Bucharest, the project aimed to promote various cognitive education strategies (e.g., making mind maps, visualization, association, mnemonics, using clues in reading comprehension, underlining keywords, scanning, self-testing, and monitoring) and various self-directed pedagogical methods (e.g., cognitive gamification-based learning and cognitive problem-based learning) for different educational levels (e.g., kindergartens, primary school, and secondary school) of children with learning difficulties or in the management of particular behavioral difficulties.

The pandemic period revealed the harsh reality of the Romanian educational system. More than a third of Romania's children are at high risk of poverty, and a significant number of them come from rural areas where the support educational services (offered by support teachers) are almost non-existent. In 2020, Eurostat ranks Romania 1st in the EU in terms of the risk of poverty/social exclusion, child poverty being higher in children (41.5%) than in the general population. Even by the OECD definition, disadvantaged students are included in the C category of special educational needs; in Romania, the service of support teacher is only offered to children with disabilities. It is difficult to outline, statistically at a national level, the problem of schooling children with special needs in rural areas and the support services provided to them, due to the general lack of transparency or concern at the level of the relevant ministries (Eurostat, 2021).

Romania's situation regarding SDG1 (no poverty), in correlation with SDG4, highlights the context in which children with special needs entered the educational lockdown.

The Romanian educational system has been plagued for many years by an increased dropout rate (between 16.4 and 19.1% in the last 10 years) and functional illiteracy (between 38 and 42% in the last 10 years), Save the Children (2019) which places it at the bottom of European rankings. Romania is in a situation of a real educational crisis since many students from rural or urban disadvantaged schools do not have the necessary devices or the necessary skills to use them.

In this context, TactileImages becomes an important tool for distance education and for independent development. Based on interactive augmented reality and in the context of the CORTTEX project, TactileImages creates personalized educational materials in an interactive audio self-described manner.

## SDG Commitment During the Pandemic Period and the New Lens for Inclusion—Tactileimages and CORTTEX Contribution to Achieve the SDG Vision in Therapy and Inclusive Education

In Romania, due to the chronic lack of support for research in special education for over 15 years, we only have small-scale initiatives developed by small groups on isolated topics. Although the Ministry of Education has permanently collected information from schools during the pandemic, it has not yet brought forth a document for a diagnosis of the situation in special education (especially in rural areas and for poor families) or proposed measures for improvement. All reports are made by international organizations such as UNICEF, which are conditioned financially. For example, Save the Children periodically monitors the children's rights in Romania, but only on fundamental rights. We specify that the Romanian authorities do not monitor the rights of people with special needs, and there is no national or international reporting.

That is why we will focus on international reports made during this pandemic period and especially on the solutions they propose.

The World Bank report “Pivoting to Inclusion: Leveraging Lessons from the COVID-19 Crisis for Learners with Disabilities” (World Bank, 2020) presents recommended practices for educational and social inclusion and participation for children with disabilities. In this context, specialists have to rethink education with the inclusive lens of the pandemic period, and its central action is remote learning.

Two challenges are identified about remote learning: one is about the systemic inequalities expressed by accessibility, ability, and affordability, and the second challenge is about designing remote learning, to which the authors propose solutions such as Universal Design for Learning, which engages the learners to think, develop skills, and grow while at home.

The lessons learned from the COVID-19 are valuable in understanding the limitations of educational service systems, emphasizing the need for a change of perspective (Basham et al., 2020). In this context, in countries where education is not really a priority of the government, the question is how we should rethink these educational services considering the evolutions in technology. Technology, in all its forms, is part of our lives—at home, at school, at work. In the pre-pandemic period, online learning tools were used not only in classrooms but also at home, as complementary ways to increase the attractiveness and interactivity of learning experiences or to provide knowledge and skills. The use and usefulness of technologies have been scientifically validated for children with special needs, as a significant support for enhancing their cognitive and emotional development in schools. Especially for children with autism spectrum disorders (ASD), Carrington et al. (2020) today there are multiple behavioral modeling attempts through virtual learning environments. In interaction with the simulated life situation, children with ASD, and not only them, can be prepared/desensitized to respond to more or less complex or unpredictable life situations by avoiding blockage and panic behaviors. These models can use conversation agents, relational agents, pedagogical agents, and chat-bot agents (Ramachandiran et al., 2015). Virtual learning environments can contribute significantly to cognitive and behavioral therapy. As Papoutsi et al. (2018) highlight, emotion-aware apps have a significant contribution to improve emotional intelligence and a better integration into social contexts for people with ASD, to understand the mind of others and to be better understood by their caregivers, educators, and parents.

We already know the importance of assistive technology (AT) in increasing the quality of life for people with special needs. Koch (2017) considers computer-embedded AT as a component of universal design (even for learning), so using it teachers facilitate students' learning creating more independent, more motivated students who have greater access to their curriculum.

Remote teaching is one of the solutions that the pandemic has highlighted. Specialists differentiated the “online learning experiences generated by the pandemic” from the “online learning” as a term theorized and developed before the pandemic as “planned from the beginning and designed to be online” (Hodges et al., 2020).

In this context, TactileImages becomes an important tool for distance education and for independent development.

Structural TactileImages includes the following:

- Image Creator is an innovative software that allows the creation or adaptation of usual images as interactive tactile vectorial drawings that self-describe *via* audio output and can be explored independently by any person (universal design) with the help of a mobile app. Image Creator has three main components, namely, the Drawing Tool, the Editor, and the Map. The first is used to create drawings from scratch, the second allows adding descriptions to any drawing, and the last one allows creating a map of one's surroundings.

The Drawing Tool offers different functions such as a) free drawing (create predefined shapes, add text and color, and also use the pencil to draw freely); b) clipart (there is an entire library of clipart you can use to create drawings easily by drag and drop your favorite elements, but you can also upload your own clipart); and c) background (this function allows turning any picture into a drawing easily).

The Editor adds captions to drawings or pictures. We can add as many words as we want to any part of the drawing or picture. The descriptions work just like the indications given by the teacher, in a non-linear mode. So, children can explore the tactile images independently with the help of a mobile application (currently available only for iPhone, but it will be developed for the Android version in the future). Using algorithms of artificial intelligence, machine learning, finger tracking and interactive augmented reality, the latter identifies the place where the finger is positioned on a tactile graphic with an attached QR code, and the information is then transmitted *via* Voiceover (a screen reader in which auditory descriptions help you navigate easily with a Bluetooth keyboard or simple gestures on a touch screen or trackpad).

The connection between the physical board and the application is made through a QR code. The QR code facilitates information input in specific areas of drawn space, which provides educational and therapeutic interest and allows the personalization of tasks.

The Tactile Images READER App does not require human interaction because it can play the role of the person assisting the child with special needs, explaining to him or her what he or she has under the finger, thus creating a coherent representation of the drawing. The App works only when the phone is placed above tactile graphics.

Based on interactive augmented reality, TactileImages creates personalized educational materials in an interactive audio self-described manner in the framework of the CORTTEX project.

CORTTEX curriculum is based on a needs analysis (Folostina et al., 2021); 600 specialists (therapists and teachers) from 3 countries were involved (i.e., Romania, Belgium, and Greece). The research methodology used was a mixed cross-sectional methodology (quantitative and qualitative), which provides a comprehensive perspective on several aspects regarding the therapeutic intervention and interaction for students with learning difficulties (LD): attitude and knowledge, concerns about teaching students with LD, methods for identifying students with LD and assessing their cognitive skills, integrated curriculum use, methods used for cognitive therapy, management of challenging behaviors, parents relationship, methods of enhancing empathy in children with LD, and education quality. Specialists analyze the nine-layer pyramidal model on emotional intelligence in order to improve the CORTTEX curriculum (Drigas and Papoutsi, 2018).

CORTTEX curriculum is structured in three dimensions as shown in Table 1.

**Table 1:** CORTTEX curriculum structure.

**Preschool level**		
**Basics**: color, shape, size, spatial orientation (with spatial landmarks), number and quantity (1-10), human body (body parts), time (unconventional instruments, observable aspects: day-night), relationship cause-effect (with intuitive support), basic emotions (joy, sadness, fear, anger)	**Adaptive behaviors**: Motor skills (fine and coarse), autonomy in personal hygiene, getting dressed, feeding, age-specific household activities, general knowledge about the world (family environment), communication (e.g., use of a minimum vocabulary of politeness: greeting, Please, Thank you)	**Cognitive behaviors**: reaction to stimulus, concentration, imitative behaviors, question and answer formulas, symbolic play, initiation / adaptive play
**Primary level**		
**Basic concepts**: color, shape, size, spatial orientation (with the relevant language), numbers and quantity (1-1,000,000), human body (body parts and the way they work), the time (telling the time), cause-effect relationships (identified in given contexts), basic emotions (joy, sadness, fear, anger, boredom, amazement, etc.)	**Adaptive behaviors**: motor skills (fine and coarse), autonomy in personal hygiene, getting dressed, eating routine, age-specific school and household activities, general knowledge about the world (family/school/play environment), emotions management, social behaviors: interrelationships, observance of rules, good manners	**Cognitive behaviors**: reaction to stimuli, concentration, imitative behaviors, question and answer formulation, symbolic play, initiation / adaptive play, concept formation, correct perception of a situation, decoding a verbal message, problem solving (age-appropriate contexts), use of nonverbal ways in communication, generalization and abstraction (as far as possible without intuitive support)
**Lower secondary level:**		
**Basic concepts**: spatial orientation (verbalization, use of verbal labels), cause-and-effect relationship (identified in personal life contexts), basic emotions (joy, sadness, fear, anger, boredom, amazement, frustration, disappointment, etc.)	**Adaptive behaviors**: age-relevant autonomy in school and household activities, involvement in community activities, general knowledge about the world (family/school/community), emotions management, social behaviors: interrelationships, observance of rules, good manners, volunteering	**Cognitive behaviors**: reaction to stimuli, concentration, formation of concepts, conservation and constancy, correct decoding of a situation, decoding a verbal message, problem solving (age-appropriate contexts), use of nonverbal ways in communication, generalization and abstraction without intuitive support), abstract / integrative thinking, use of tools for memory development, focus on task

The second part (Part II) of the curriculum brings a higher dimension of cognitive processing, offering the perspective of “thinking skills.” Part II is aimed at primary and secondary schools.

Mitsea et al. (2021) highlight that training in adaptability and flexibility in various domains (cognitive, emotional, and behavioral) is very important for students to be better prepared to adjust to uncertainty and to rapid change (Drigas and Papoutsi, 2021). An appropriate training in cognitive and socio-emotional skills can integrate them in an academic, working, and social environment, but this can happen only when we train the metacognitive skills. The authors conclude that soft skills are fully trainable and are entirely dependent on the development of metacognitive skills even if, initially, the literature considered that soft skills have a rich cognitive background (led by attention, working memory, and other executive functions).

Focusing on the CORTTEX curriculum and using TactileImages tools, specialists (psychologists and psycho-pedagogues) created examples of curricular sets (in fact therapeutic programs for different areas of development—perceptual, cognitive, emotional, and social, behavioral) with all the benefits of remote teaching.

These sets of materials illustrate the ways in which the therapeutic approach is conducted and offer the premises for its personalization. The functioning profile justifies the manner of personalization in the heterochrony of the student's individual development.

The resources are designed for therapists and teachers, for families, and even for children, if they use them under professional guidance. Children can study the drawings independently (or as independent work) only with the help of the assistant app that reads the descriptions just like a teacher (even using a personalized voice in the future features of the e-learning platform).

## Remote Teaching—the Deployment of Therapeutical and Educational Personalization Process Using CORTTEX Curriculum and Tactileimages Tools

In order to attain the goals of SDG 4, it is necessary to personalize the educational and therapeutic intervention. The term “personalized learning,” although recommended, is often less methodologically defined, but the wide range of approaches broadly seeks to tailor the content, support, and pathways that students learn (Alli et al., 2016).

Researching the impact of the COVID-19 pandemic on learning in Germany in terms of perceived self-efficacy of teachers working with children with special educational needs, Maurer et al. (2021) highlight the low values of this important motivator for the teachers' activity. This seems to result from challenges in the identification of difficulties and support offered in distance learning to the students with difficulties in reading, writing, and mathematics due to a lack of use of concrete materials.

Unlike other e-learning platforms, TactileImages uses concrete material and does not transpose the child into a virtual world, but brings digital advantages to the real world. Tactile images (with embossed areas), as the main mediators of knowledge in this approach, are not only useful for visually impaired children but also bring focus and essential information to children with autism, attention disorders, and mental deficiency and, last but not least, to children without difficulties. For this reason, we can say that TactileImages offers premises for a universal design of learning. Universal design for learning is a framework that supports the design of a learning environment and accepts the variability of every learner (Nelson and Basham, 2014). The same authors see teachers when they put this framework into action as “learning engineers” because they are designers of development solutions “focused on overcoming barriers through a process of problem-solving and iterative design.”

We can only plan or attain an educational goal with a good understanding of individual psychological characteristics and mechanisms of learning. That is why we use TactileImages as a tool in the CORTTEX matrix to improve individual development through personalization. We consider the illustrated approach as pertaining to educational psychology.

The ICF functioning profile, as structure and content, identifies and monitors the effectiveness of intervention strategies of rehabilitation professionals; it is also an important tool for clinicians and researchers (Ustün et al., 2003). We used it as a starting point for personalization. By mapping out the level of development of specific mental functions, we can explore the functional profile to find the poorly developed areas that need intervention, and we can also highlight the compensation areas (those areas with a good level of functioning that we can use as a foundation/anchor during intervention).

CORTTEX provides the necessary content elements, and TactileImages provides the intervention tool. We will exemplify some specific ways of intervention for the cognitive development of various categories of children—we will illustrate the contribution of Tactile and CORTTEX for training life skills in verbal and non-verbal children. In the following, we show morning activities where Tactile organizes a sequence.

**Figure 1:**
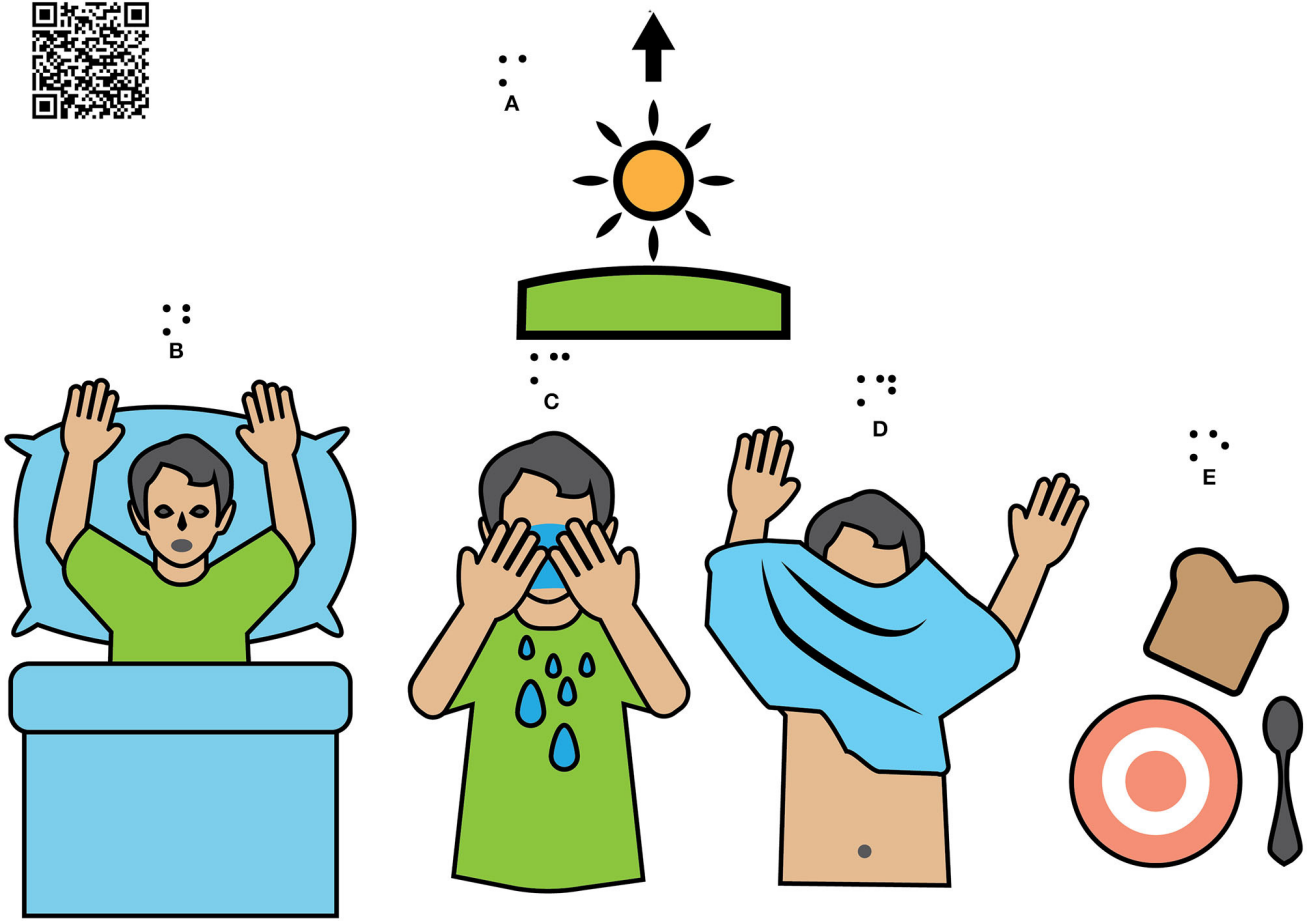
Sequencing the day's activities.

On the poster in Figure 1, information on various levels of complexity can be attached to the QR Code fields. When the finger touches the QR area/picture for at least 3 s, it triggers the rendition of a prerecorded message. Recorded messages will soon be available so that the child can hear familiar voices (mother, teacher, support teacher, etc.) to improve the quality of remote teaching.

The posters below are designed for children who have difficulties in building daily routines, and they can also be used as an educational material for the development of complex perception of time, order, and succession. Preschoolers without learning difficulties can benefit from this educational material too.

For example, the morning icon (sun up) carries different messages appropriate to the child's level of understanding from a simple “Good morning, Daniel!” to a more elaborated: “Good morning, Mihai! The sun is up and brings you a wonderful day!” / “(It's a wonderful day!)”.

The icons in row 2 carry a QR-coded message with verbs in the simple present tense, first person:

“I wake up/get out of bed, wash my face, get dressed, eat.” or can share a story about personal hygiene including details about the instructions needed to guide the child for the day.

The same poster can carry another personalized message (by QR) that the specialist considers suitable for another child or by matching the level of complexity to the growing learning needs of the student from one lesson to another about one topic.

The pictures are printed in relief, so tactile exploration brings extra information to the visual image, which improves the attention span. It can also be used by visually impaired children, and we consider it to be an educational material that respects the principles of universal design for learning. The icons were taken from arasaac.org, but were edited to suit the educational context.

It is a learning support not only for reading the dial clock but also for understanding the concept of night-day, darkness-light. Together, they are part of a set proposed by CORTTEX specialists which aims at both adaptive behaviors and learning specific concepts.

Adaptive behaviors (basic skills related to daily living) such as getting dressed, personal grooming and physical self-care, eating and table manners, orientation in the environment, helping in home activities, and general knowledge about the immediately experienced world are plans with sequenced activities.

Reader App allows the child to return to the previous sequence of activities, including separate exploration, and fix them. The QR code allows the task to be more complex as the child learns the basics. The QR code is, in fact, the one that ensures the personalization of these tools.

Basic concepts such as color, shape, size, orientation in space, number and quantity, time, cause and effect relationships, feelings and moods, and the human body (body parts and their functions) can be illustrated in sets of specialized drawings.

All CORTTEX-developed materials can be downloaded free of charge from the section https://tactileimages.org/ro/category/cortex/.

They can be printed using affordable techniques from tactileimages.org. As to the various functions of TactileImages, personalized tactile pictures can be attractive even in a post-pandemic context as they help diversify learning tasks by using shared visual support for all the students in a class.

The TactileImages platform becomes an alternative pedagogical solution that contains, in addition to the mobile application, a virtual library of 1,000 self-describing drawings that can be added simply *via* drag & drop when teachers or parents plan personalized educational resources.

Teachers and parents can access or adapt these resources with personalized descriptions and use them as a function of the child's learning dynamic.

In conclusion, we consider TactileImages as an e-tool for universal design curricula because it fulfills the principles of UNESCO's well-established good practices for inclusion and equity such as:

- *Clarity of meaning*: The design of educational materials takes into account the diversity among learners since the very beginning.
- *Analysis of context*: With personalized tasks and remote teaching, it also contributes in the pandemic context to the improvement of the child's status (the functional profiles offer a psychological perspective for the therapeutic and educational approach) aiming to increase participation and achievement.
- *Building on existing practices*: The platform improves the existing educational offer and the possibilities of cooperation and free access, thus giving the opportunity to transfer expertise within and between schools.
- *Working collaboratively*: Not only does it offer the potential for collaboration, but it also emphasizes the importance of promoting mutual support among stakeholders to shift practices; the child and parents can give important feedback to the specialist in personalizing the therapeutic and educational approach.
- *Managing change*: Being an innovative tool, it brings about change in the therapeutic approach, including at the classroom level. It facilitates the reduction of frequent bureaucracy in case management and makes the process more efficient.
- The evaluation of progress is easier to record and test, as this practice focuses on the implementation of the bio-psycho-social model, and the impact of change is recorded in the functioning profile (UNESCO, 2021).

All the benefits that the implementation of UDL instruments has at local (schools) and national levels are illustrated in the paper A blueprint for UDL. Considering the design of implementation (Nelson and Basham, 2014), we list the ones that are very important for a country like Romania according to the illustrated socio-educational context:

- Therapists/teachers will have a better understanding of instructional strategy and design, instructional technology, cognition and learning, proactive behavior management and student engagement, and self-determination.
- Therapists/teachers will be data-driven problemsolvers and active designers of teaching as an iterative process influenced by learner variability and performance.
- Therapists/teachers will connect instructional resources and technology infrastructure, technology being simply another tool or resource for supporting teaching and learning.
- Encourage therapists/teachers to innovate and iteratively design around barriers to learning.
- Often, design models of instruction are actively communicated and exchanged for sharing ideas and developing solutions with other colleagues' expertise as a supported culture of idea and resource exchange among different educational organizations and specialists.

## Discussions on the Practical Advantages Offered by Tactileimages as a Personalized Learning and Therapeutical tool for the Peri And Post-Pandemic Period

During the development of TactileImages as an innovative educational solution, the needs identified in relation to the beneficiaries and in relation to the Romanian educational system were permanently taken into account.

**Figure 2:**
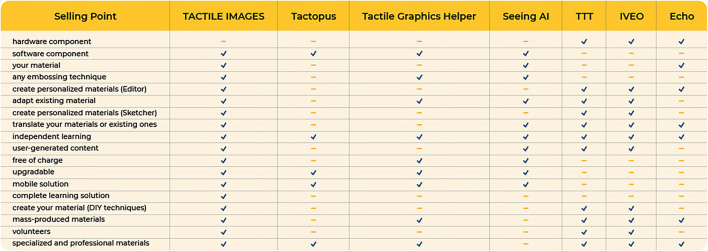
Benchmarking for tactile images.

The defining characteristics for TactileImages compared with the main identified competitors are given as follows (Figure 2).

- Tactopus is a creator of interactive books, games, and puzzles that can be explored with a digital assistant. https://tactopus.com/.
- Tactile Graphics Helper is a mobile app that tracks the user's finger as they explore a tactile graphic, announcing information about the location they are pointing to. https://apps.apple.com/us/app/tactile-graphics-helper/id1469997677.
- Seeing AI is mobile app that identifies persons and objects and describes them *via* audio output. https://www.microsoft.com/en-us/ai/seeing-ai.
- TTT or The Talking Tactile Tablet is a computer peripheral device designed for use as a “viewer” for audio/tactile materials. http://touchgraphics.com/portfolio/ttt/.
- IVEO allows users to understand tactile graphics in an engaging and interactive solution. https://viewplus.com/product/iveo-3-hands-on-learning-system/.

Thus, milestones were outlined, elements that we used in the benchmarking process in the analysis of competitors, such as:

- *Hardware component*: TactileImages working on creating 4 tactile printers based on wood-glue.
- *Software component*: TactileImages includes a mobile app and drawing software.
- *Personalization*—Create educational materials (using the Editor/ Sketcher) or adapt existing material: Anyone can write their own descriptions or add descriptions to the already created materials to be suitable to the age and knowledge level of the beneficiary.
- *Embossing technique*: Anyone can add audio descriptions to any tactile material, regardless of the embossing technique.
- *Translate materials or existing ones*: Anyone can translate the descriptions of the tactile graphics in the library into their own language.
- *Independent learning*: Anyone can use the mobile assistant to study interactive tactile graphics independently.
- *User-generated content*: Users are free to create their own materials.
- *Free of charge*: Anyone can access TactileImages e-learning platform (library, drawing software, mobile app) for free.
- *Upgradable*: Automatically.
- *Mobile solution*: Anyone can use their own mobile app to study at school, at home, in the park, train station, museum—wherever there is a stable internet connection.
- *Mass-produced materials*: Any drawing can be reproduced on a wider scale.
- *Specialized and professional materials*: Tactile catalogs are created with the help of teachers mainly from special schools.

There are software solutions (apps) on the market which can identify objects and describe them audibly for multisensorial learning purposes. They use image recognition algorithms, but do not offer additional information, they just recognize the object (e.g., the apps utter: elephant, whale, mug). The disadvantage is that the descriptions cannot be personalized, according to the specific needs of every child/lesson requirement, and do not offer detail, representing just a general description (Patzelt and Blaj, 2020).

In our perspective, TactileImages represents a necessary e-tool for personalizing the educational and therapeutic intervention not only for the pandemic period but also for the post-pandemic period, marking a shift in the evolution of the field and generating an important progress for ensuring the quality of inclusion.

In our qualitative study, we involved 10 specialists (5 teachers and 5 therapists, 7 of them tested TactilesImages in the special schools and 3 in the mainstream schools) using diagnostic teaching research methodology to collect data about the educational direct impact of 5 custom drawings made using TactileImages and the CORTTEX curriculum. Observation grids were based on the functioning profile of the student, to which Likert scales 1–10 were attached (according to Table 2, we selected the most impacted functions).

**Table 2:** Body functions improvement.

**Body function**	**Observed improvement interval**
b1140 Orientation to time	1 to 7
b1141 Orientation to place (TactileImages space)	3 to 10
b1142 Orientation to person	2 to 7
b1264 Openness to experience	4 to 10
b130 Energy and drive functions (G)	3 to 10
b1304 Impulse control	3 to 8
b1400 Sustaining attention	3 to 10
b1442 Retrieval of memory	4 to 8
b1470 Psychomotor control	3 to 10
b1471 Quality of psychomotor functions	3 to 10
b152 Emotional functions (G)	2 to 7
b1560 Auditory perception	4 to 10
b1561 Visual perception	3 to 10
b1564 Tactile perception	5 to 10
b1565 Visuospatial perception	3 to 10
b1643 Cognitive flexibility	1 to 5
b16700 Reception of spoken language	2 to 8
b4552 Fatigability	(−1) to (−4)

**Figure 3:**
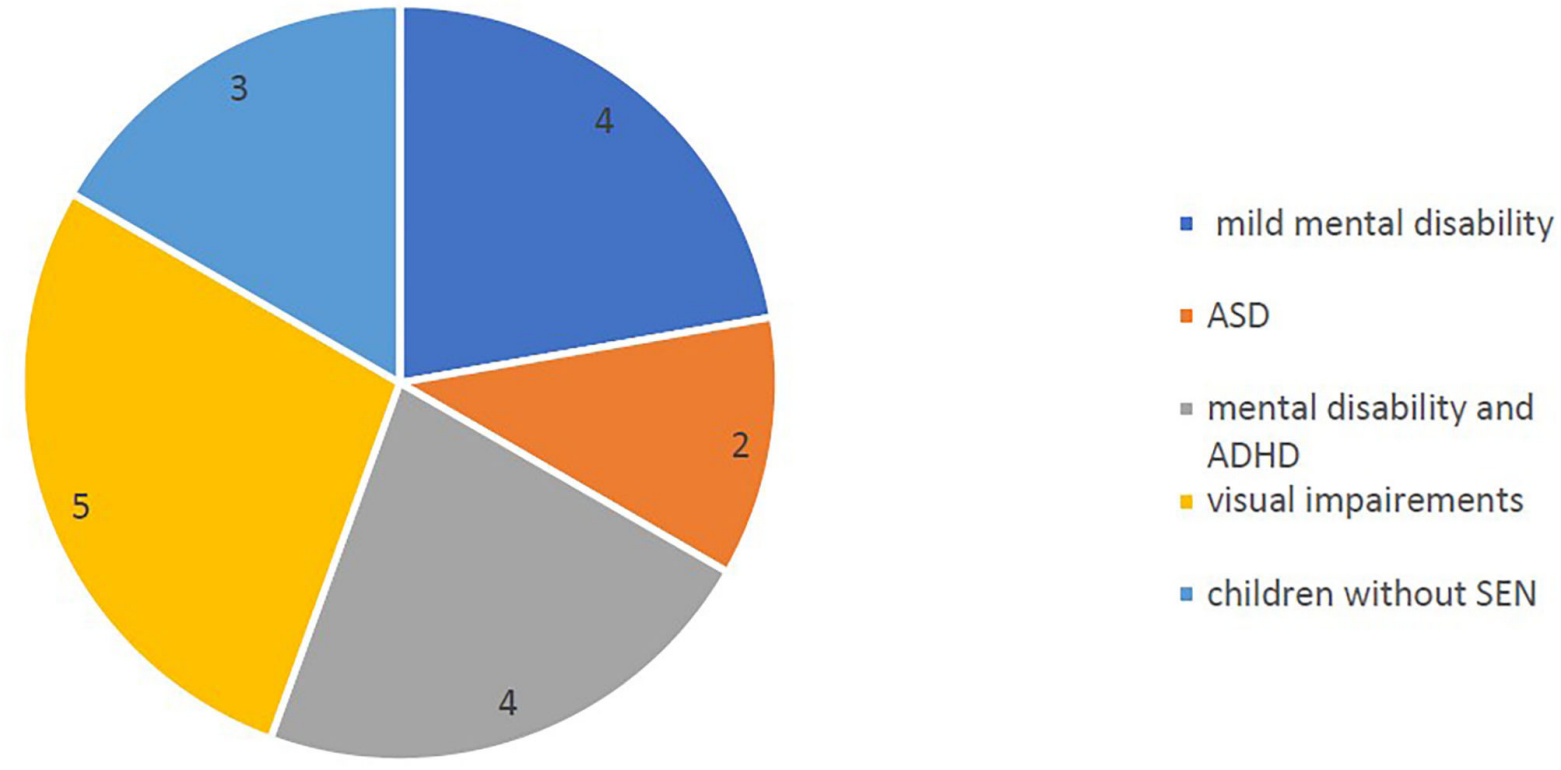
Categories of students observed.

The 18 children observed belong to the categories presented in Figure 3.

We note that the most intense impact was recorded on the following:

- *b1564 Tactile perception* (improvements recorded from 5 to 10 points), more precisely on the use of tactile information in educational tasks.
- *b1264 Openness to experience* (improvements recorded from 4 to 10 points) due to the attractiveness of multisensory information recorded for all children observed.
- *b1560 Auditory perception* (improvements from 4 to 10 points) to complete visual/tactile information.

Other functions with notable improvements (from 3 to 10 points) were recorded on b1141 Orientation in the sheet's space, b130 Energy and drive functions, b1400 Sustaining attention, b1470 Psychomotor control, b1471 Quality of psychomotor functions, b1561 Visual perception, and b1565 Visuospatial perception.

From the perspective of the intervention, the specialists observed hierarchical improvements (by frequency): increase the attractiveness of the activity and the level of involvement of students in the task (100%), a better relationship with the one who facilitates learning (80%), increase the capacity of intellectual effort (80%), a better understanding of abstract concepts (50%), and understanding of emotions (50%). From the perspective of personalization, all the specialists involved appreciated the possibility offered by the platform to create their own images and to adapt the content according to the needs of each student through the facilities offered by the QR code.

We could say that the role of CORTTEX and TactileImages is not only to improve the results of SDG4 but also to reduce the effects of SDG1.

## Acknowledgment of Any Conceptual, Methodological, Environmental, or Material Constraints

This article illustrates the evolution of an educational solution dedicated to the creation of personalized educational and therapeutic material as a pandemic response brought to the Romanian educational system challenges and also to the Sustainable Development Goals Agenda (Arthars et al., 2019).

Although we did not benefit from a diagnosis and a report from the authorities about the education of children with special needs during the pandemic, by referring to educational systems with a higher commitment regarding SDG goals, the authors brought improvements to the educational solution. In this context, they completed this personalization tool with remote teaching functions, which are considered necessary for this period. The approach is qualitative and brings to the fore how this tool has evolved and how it can contribute to the curriculum developed within the CORTTEX project, a project whose results are based on the principles of neuro-didactics.

Some online tools shift could change teachers' roles, making them more like coaches and mentors (d'Orville, 2020).

## Data Availability Statement

The raw data supporting the conclusions of this article will be made available by the authors, without undue reservation.

## Author Contributions

LP, RF, and BP: curriculum development and applied study. DP and MB: technical development and description of the platform design. All authors contributed to the article and approved the submitted version.

## Funding

This work was funded by the budget of the Erasmus + project Cognitive Resources for Toddlers Teens and Experts—CORTTEX - 2020-1-RO01-KA202-080239.

## Conflict of Interest

The authors declare that the research was conducted in the absence of any commercial or financial relationships that could be construed as a potential conflict of interest.

## Publisher's Note

All claims expressed in this article are solely those of the authors and do not necessarily represent those of their affiliated organizations, or those of the publisher, the editors and the reviewers. Any product that may be evaluated in this article, or claim that may be made by its manufacturer, is not guaranteed or endorsed by the publisher.
